# Rosemary Extracts Improved the Antioxidant Status of Low-Fat Yoghurt Sauces Enriched with Inulin

**DOI:** 10.3390/antiox11040789

**Published:** 2022-04-16

**Authors:** Magdalena Martínez-Tomé, Cristina Cedeño-Pinos, Sancho Bañón, Antonia M. Jiménez-Monreal

**Affiliations:** 1 Department of Food Science, Veterinary Faculty, Regional Campus of International Excellence “Campus Mare Nostrum”, University of Murcia, 30100 Murcia, Spain; antoniamjimenez@um.es; 2 CIBER, CB12/03/30038 Fisiopatología de la Obesidad y la Nutrición, CIBEROBN, Instituto de Salud Carlos III (ISCIII), 28013 Madrid, Spain; 3 Department of Food Technology and Science and Nutrition, Veterinary Faculty, Regional Campus of International Excellence “Campus Mare Nostrum”, University of Murcia, 30100 Murcia, Spain; cristinacarmen.cedenop@um.es (C.C.-P.); sanchoba@um.es (S.B.)

**Keywords:** inulin, rosemary extract, yoghurt sauce, antioxidant, polyphenol

## Abstract

Yoghurt sauces are considered fatty products which are quite susceptible to oxidation and must be stabilised using antioxidants. Novel formulations for yoghurt sauces often involve replacement of fat with dietary fibres and use of natural preservatives. The aim of the present research was to design healthier formulations for yoghurt sauces based on the replacement of sunflower oil (SO) with chicory inulin (IN) and the use of rosemary extracts (RE) as natural antioxidants. Different sauces were developed by adding IN at 2 and 5% w: w and/or 300 mg/kg lipo- and/or water-soluble rosemary extracts (RLE and/or RWE) containing 120 and 146 mg polyphenols per g extract, respectively. Nutritional value (proximate composition and caloric contribution), some physical properties (pH and CIELab colour) and antioxidant status (deoxyribose, DPPH radical scavenging, Rancimat, lipid peroxidation and linoleic acid assays) were assessed in the sauces. Replacement of SO with IN (5%) reduced fat content by 30%, roughly 15% low calories, thereby obtaining healthier sauces. As expected, the RLE was more effective than the RWE in improving antioxidant activity in lipidic environment. Using RLE enhanced the antioxidant capacity of lipid peroxidation by 44%. In the Rancimat test, this increased the oxidative protection of the sauce made with and without IN (5%) by around 20% or 45%, respectively. Similarly, using RLE doubled protection against linoleic acid oxidation. Application of IN in yoghurt sauce has nutritional (replacement of lipids with dietary fibre) and technological interest (foaming agent) and can be combined with RE of high polyphenol content as a potential functional ingredient capable of stabilising the sauces against oxidation.

## 1. Introduction

Nowadays, lack of time for cooking and demand for comfort and speed of food production have brought an increase in the need for precooked dishes, in which sauces play an important role [1].

According to the Codex Alimentarius, the category of salts, spices, soups, sauces, salads, and protein products includes substances added to foods to enhance aroma and taste. The sauces and condiments category is dynamic, with large differences in consumption habits and practices among countries [2].

In the United States, commercially produced sauces are not legally defined except for mayonnaise, spoonable salad dressing, and French dressing. Traditionally, these foods have been classified as acid foods with a pH below 4.6. In general, these products comprise a vegetable oil or a fat-free oil substitute base and a low pH, salt-containing water phase [3].

Due to their composition, sauces are considered more susceptible to an oxidation process; therefore, they are widely studied and evaluated regarding their antioxidant behaviour and often compounds with antioxidant activity are added that prevent early oxidative degradation.

In recent decades, the addition of natural antioxidants to foods to prevent oxidation is more widespread. Rosemary plants (*Rosmarinus officinalis* L.) have long been used due to their powerful antioxidant and antimicrobial potential [4]. Recently, the European Union admitted the use of RE as a natural antioxidant (E-392) in different food products by adding it with a maximum of 300 ppm of carnosic acid and carnosol [5]. There are three types of rosemary extract: whole, RWE (rich in rosmarinic) and RLE (rich in carnosic and carnosol). These three phenolic diterpenes are the components identified as being mainly responsible for antioxidant activity [6].

Some researchers have observed in dairy products that RE can have an antioxidant effect, such as in butter by slowing down the oxidation process [7,8] or inhibiting lipid oxidation in cow milk enriched with fish oil [9]. Similarly, the addition of RE increased the antioxidant potential of ghee as regards radical scavenging activity [6].

Inulin is a water-soluble storage polysaccharide and belongs to a group of indigestible carbohydrates called fructans. Inulin has achieved GRAS status in the US and is widely available in approximately 36,000 plant species, among which chicory roots are considered the richest source. Inulin is commonly used as a prebiotic, fat substitute, sugar substitute, texture modifier, and for the development of functional foods even achieving a beneficial effect at the gastrointestinal level [10]. The low-fibre Western diet has contributed to an increased risk of weight gain, inflammation, chronic diseases and cardiometabolic diseases [11,12].

Native or medium-chain inulin which is present in chicory has a polymerisation degree (DP) ranging from 3 to 60 (average DP= 10–12) monosaccharide units, while long-chain inulin has from 10 to 60 (average DP = 25). The higher solubility and sweetness are detected in the short chain fraction inulin improving palatability. When inulin is used in gel form in water, it achieves better emulsions with characteristics similar to those obtained with fats [13].

Previous studies have been conducted with some success, to improve the physical and sensorial characteristics of new formulations of sauces by adding different functional ingredients, such as microcrystalline cellulose to increase fibre on white sauce [1]; low-fat with inulin white sauces [14]; inulin seems particularly suitable for fat replacement in low fat cheeses improving mouthfeel [13]; whey protein concentrate or albumin to minimise yoghurt syneresis in Tzatziki, one of the most popular Greek yoghurt-based salads [15]; or inulin added to a yoghurt as a suitable ingredient to increase dietary fibre consumption and as low-calorie alternative sweeteners in ice cream [16,17,18].

The aim of the present research was to design healthier formulations for yoghurt sauces based on the replacement of sunflower oil with inulin and/or the use of different rosemary extracts such as natural antioxidants. Nutritional value, some related properties (pH and CIELab colour) and antioxidant status were assessed in the sauces.

## 2. Materials and Methods

### 2.1. Experimental Design

A randomised statistical design with seven treatments (sauce formulations) was developed for the experiment: (1) Control; (2) IN2: inulin x sunflower oil at 2%; (3) IN5: inulin x sunflower oil at 5%; (4) RWE: rosemary water extract (300 mg/kg); (5) RLE: rosemary lipo extract (300 mg/kg); (6): RWLE: 300 mg/kg RWE + RLE (at 1:1 *w*:*w*); and (7): IN5 + RWLE. Sample size was 6 per treatment. A simple ANOVA was used to determine the effects of treatments (IN and RE) on the dependent variables. Control sauces were separately compared with the IN, RE and IN5 + RWLE sauces to determine the respective effects. The Tukey homogeneity test was used to compare the group means (*p* < 0.05). Data were analysed using the Statistix 8 for Windows (Analytical Software, Tallahassee, FL, USA). 

### 2.2. Chicory Inulin

Chicory inulin (Orafti^®^ HSI, Barcelona, Spain) was described by the supplier as a white to slightly yellow fine powder, with a slightly sweet taste. The compositional declarations were: dry matter DM: 97%; fibre: 88% DM (degree of polymerisation higher than 3); and sugars: 12% DM (glucose + fructose + sucrose).

### 2.3. Rosemary Extracts

Rosemary plants collected in summer were purchased from a local supplier and dried at room temperature. Three successive extractions were applied: (i) dried plant material (ground leaves, flowers and small stems) was distilled with hot water steam to obtain the essential-oil-containing volatile phenols (e.g., carvacrol and eucalyptol); (ii) oil-free by-product was treated with water (35 °C) to obtain the RWE containing hydrophilic polyphenols (e.g., rosmarinic acid); and (iii) the resulting rosemary by-product was treated again with acetone:water (1:1 *v*/*v*) (35 °C) to obtain the RLE containing lipophilic polyphenols (e.g., carnosic acid and carnosol). Both RE (RWE and RLE) were yellow-greenish powders with an intense flavour described as herbal, bitter and astringent. RWE was more bitter and greenish than RLE. The polyphenol content of extracts was determined using a HPLC-1200 Series (Agilent, Waldbronn, Germany) equipped with a G1311A binary pump and a G1315A photodiode array UV/Vis detector. A Zorbax SB-C18 reverse phase column was used (4.6 × 250 mm^2^, pore size 0.25 µm), preceded by a (Zorbax SB-C18 pre-column (4.6 × 125 mm^2^, pore size 0.25 µm), both from Agilent Technologies, USA. The mobile phases were acidified water (0.05% formic acid) (channel A) and 100% acetonitrile (channel B). Elution gradient was: 0 min, 5% of B; 10 min, 15% of B; 30 min, 25% of BA; 35 min, 30% of BA; 50 min, 55% of BA; 55 min, 90% of B; 70 min, and 100% B. The flow rate was 1.0 mL/min. Detection wavelengths were 280 and 330 nm. The standards were provided by (i) Sigma-Aldrich: Luteolin-7-*O*-β-Glucoronide (CAS 29741-10-4); Cirsimaritin (CAS 6601-62-3); Genkwanin (CAS 437-64-9); Hesperidin (CAS 520-56-3); Luteolin (CAS 491-70-3); 7-methyl-rosmanol (CAS 113085-62-4); Carnosol (CAS 5917-80-2); Carnosic acid (CAS 3650-09-7); 12-*O*-methylcarnosic acid (CAS 62201-71-2); (ii) Acros Organics: Ferulic acid (CAS 1135-24); Caffeic acid (CAS 331-39-5); (iii) Fluorochem: Salvianic acid (CAS 76822-21-4); (iv) Fluka: Rosmarinic acid (CAS 20283-92-5); and Luteolin-7-glucoside (CAS 5373-1135). For more details, see Cedeño et al. (2020) [19]. The polyphenol content of RWE and RLE was determined before their use in sauces (see Table 1).

### 2.4. Preparation of Yoghurt Sauce

The basic recipe for yoghurt sauce was supplemented with the following ingredients: Greek yoghurt (80.39%), honey (6.43%), sunflower oil (9.65%), Dijon mustard (3.22%) and salt (0.32%). Using this recipe, all experimental formulations were prepared and tested under the supervision of six trained panellists selected from Murcia University staff. For more details on panellist training, see Cedeño et al. (2020) [19]. The criteria followed for sensory validation of the sauces included: (i) suitable balance acidity/sweetness (inulin) and (ii) absence of rosemary off-flavours (herbal, bitter and pungent). Once ingredients homogenised, the sauce was immediately placed in jars and pasteurised (at 75 °C for 22 min). Finally, yoghurt sauces were refrigerated at 5 °C until analysis.

### 2.5. Nutritional Composition Analysis

Moisture content was determined with an infrared thermobalance (Model MA 50.R, RADWAG^®^, Radom, Poland). In total, 2 g of yoghurt sauce was evenly distributed over the surface of the dishes. A temperature ramp of 5 °C/min up to 120 °C was used during reading. Fat content was determined by Gerber method according to ISO 11870:2009 [20]. Proteins were determined by the Kjeldahl method, according to AOAC method Official Methods of Analysis reference 955.04 [21]. Ash was determined according to AOAC method Official Methods of Analysis reference 923.03 [22]. Carbohydrates were determined by difference between (% fat, % crude protein, % moisture and % ash), according to FAO (2003) [23]. The energetic value was calculated according to the energetic parameter published by the European Parliament (2011) [24].

### 2.6. Physical Measurements

Colour parameters were assessed using a CR-200/08 Chroma Meter II system (Minolta Ltd., Milton Keynes, UK), with a D65 illumination pattern, 2 viewing angle and an aperture size of 50 mm. Reflectance was measured on the surface of the sample, and results were expressed as CIELAB values: lightness (L*), redness (a*) and yellowness (b*). The pH was determined by pH meter (Crison, Barcelona, Spain).

### 2.7. Assays of Free Radical Scavenging and Antioxidant Activity

To develop the antioxidant assays the yoghurt sauce was directly added in different methods and ingredients were dissolved in water at the concentrations at which they are added to produce the yoghurt sauce.

#### 2.7.1. Hydroxyl Radical Scavenging

Hydroxyl radicals scavenging was determined by the deoxyribose assay. The reaction mixture was prepared in a final volume of 1.2 mL by adding 100 μM EDTA (ethylenediaminetetraacetic acid), 50 μM FeCl_3_, 2.8 mM deoxyribose, 2.8 mM H_2_O_2_, 100 μL yoghurt sauce or ingredient solutions and 10 mM KH_2_PO_4_-KOH buffer (pH 7.4). Ascorbate (100 μM) was added to start the reaction, and the tubes were incubated at 37 °C for 1 h. The malondialdehyde resulting from the degradation of deoxyribose was heated in water (80 °C/20 min) and it developed the malondialdehyde thiobarbituric adduct. This chromogen was measured at 532 nm [19].

#### 2.7.2. DPPH Radical Scavenging

DPPH is characterised as a stable free radical by the delocalisation of the unpaired electron which contributes to the presence of a violet colouration, with an absorption at around 520 nm. A total of 1 mL yoghurt sauce or ingredient solutions was added to 2 mL DPPH methanolic solution. DPPH, after accepting hydrogen or electrons from antioxidants, becomes yellow in colour due to formation of diphenylpicryl hydrazine. Therefore, in the presence of antioxidants, DPPH decolourises, enabling a decrease in absorbance value [25].

### 2.8. Peroxidation of Phospholipid Liposomes

According to Jiménez-Monreal et al. (2009) [26] the inhibition of lipid peroxidation was determined by in vitro generation of the lipoperoxyl radicals. In a final volume of 1 mL, the assay mixtures were made up with phosphate-buffered saline, 0.5 mg/mL phospholipid liposomes, 100 µM FeCl_3_ and 100 µL of yoghurt sauce or ingredient solutions, and 100 µM ascorbate. Incubations were carried out at 37 °C for 60 min. At the end of this incubation period, 1 mL each of 1% (*w*/*v*) thiobarbituric acid (TBA) and 2.8% (*w*/*v*) trichloroacetic acid were added to each mixture. The solutions were heated in a water bath at 80 °C for 20 min to develop the malondialdehyde-thiobarbituric adduct [(TBA)_2_-MDA]. The (TBA)_2_-MDA chromogen was extracted into 2 mL of butan-1-ol and measured at 532 nm.

### 2.9. Rancimat Test for Oxidative Stability

The protection of sunflower oil against oxidation was analysed with the Rancimat (Metrohm model 743, Herisau, Switzerland) by heating at a temperature of 120 °C and an air flow of 20 L/h. Samples were prepared by mixing the yoghurt sauces or ingredient solutions with the oil (80%, *w*/*w*) under stirring for 3 h at 25 °C. The values resulting from the determination indicate the induction time, which are used to calculate the protection factor (PF) [27].

### 2.10. Determination of Antioxidant Activity in a Linoleic Acid System

To measure the inhibition of linoleic acid autoxidation, linoleic acid solution (a solution of 10 mL of linoleic acid (11.7 g/L in 99.8% ethanol) and 10 mL of phosphate buffer, 200 mM, pH 7.0) and 5 mL of yoghurt sauce or ingredient solutions were added. The total volume was adjusted to 25 mL with deionised water. This solution mixture was kept at 40 °C (adverse temperatures) for 4 weeks. The evolution of oxidative protection was measured weekly by spectrophotometry at 500 nm [27].

## 3. Results

### 3.1. Proximate Composition of Yoghurt Sauce

Table 2 shows the proximate composition of yoghurt sauce with or without inulin and/or rosemary extract. All sauces show similar composition of moisture, ash and protein. However, significant differences are found in fat content (*p* < 0.001) and carbohydrate content (*p* < 0.01) between sauces with or without inulin. When the sunflower oil ingredient was substituted for inulin 5%, fat content decreased by around 30%. These differences (*p* < 0.001) are also observed in the energy value; the sauce with the highest level of inulin (5%) showed roughly 15% lower calories than the Control sauce.

When these yoghurt sauces are compared with commercial types (Table 3), results show a very high level of fat and energy content, except for sauce number 6, which is also supplemented with yoghurt (70%) and the energy value is similar to IN5 yoghurt sauce. The remaining commercial sauce, supplemented with sunflower/soybean oil as the first ingredient and yoghurt included in a lower amount, presented the highest energy values.

### 3.2. PH and CIELab Colour

Table 4 shows data concerning CIELab colour and pH of sauces. IN5 replacement slightly decreased L* values and increased the b* value compared to the Control and IN2 samples, while a* values were similar for these three formulations. Moreover, the sole addition of RE affected the CIELab colour of yoghurt sauces, increasing the b* value. The most relevant change in sauce colour corresponded to the IN5 + RWLE formulation, which presented the lowest L* (*p* < 0.01) and the highest b* values. Thus, the combination of inulin and RE resulted in a slightly darker and yellow yoghurt sauce (*p* < 0.001).

### 3.3. Antioxidant Capacity or Status

The antioxidant activity of the different sauces was compared using the deoxyribose, DPPH, Rancimat, lipid peroxidation and linoleic acid assays. Oxidative damage can be generated by different mechanisms of action of ROS, so it is interesting to use several techniques to evaluate the antioxidant behaviour against free radicals [28].

Hydroxyl radicals are extremely reactive and may be generated under physiological conditions in the human body, where they react with non-selective compounds such as proteins, DNA, unsaturated fatty acids and almost every biological membrane [19].

**Figure 1:**
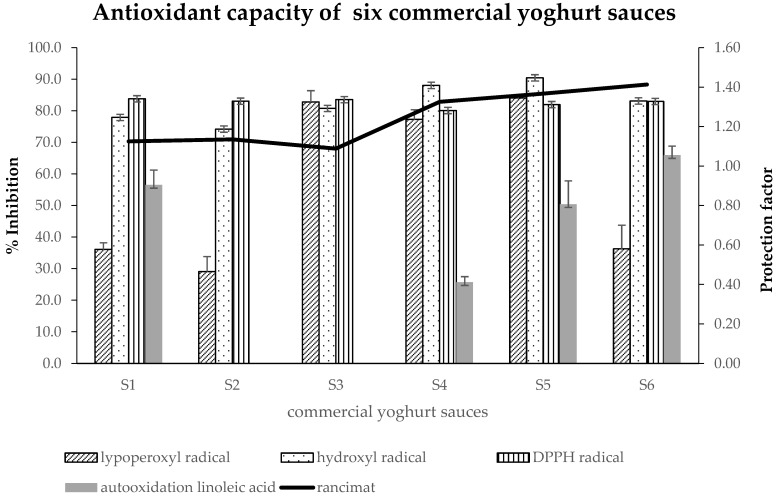
Average (*n* = 3) antioxidant activity of six commercial yoghurt sauces evaluated using different assays (lipid peroxidation, hydroxyl and DPPH radical scavenging, autooxidation linoleic acid and Rancimat test).

Table 5 shows the deoxyribose damage caused by OH radical in the presence of yoghurt sauces with or without inulin and/or rosemary extract. All sauces present a high OH· scavenger capacity, with values close to 90% inhibition. The results of ingredients show that honey (78.30%) and Greek yoghurt (74.87%) are the ingredients with the highest OH· scavenger capacity, although percentages of inhibition observed in sauces were even greater (Table 6). All sauces, Greek yoghurt, honey and Dijon mustard can be considered as primary antioxidants because when ascorbate is omitted, during the reaction, they showed lower levels of absorbance than the control (data not shown). Regarding commercial sauces analysed (Figure 1), all showed values similar to yoghurt sauces with values around 80%.

In DPPH radical scavenging activity, the antioxidant scavenges the free radical by offering electrons or hydrogen to unpaired electrons [29]. Table 5 shows that all sauces are good DPPH radical scavengers with no significant differences shown between them. The ingredients of sauces analysed at the amount used in yoghurt sauces (Table 6) show that rosemary extracts (RWE and RLE) are those with the highest value (around 90% of inhibition). The antioxidant capacity was, in decreasing order, Dijon mustard, Greek yoghurt, honey, inulin, salt and sunflower oil, all with inhibition percentages of between 79% and 35%. In the case of commercial sauces (Figure 1), values observed were around 80% of inhibition.

Table 5 shows that lipo-soluble rosemary extract enhanced the antioxidant capacity (increased 44%) for the three yoghurt sauces (RLE, RWLE and IN5 + RWLE), with significant differences (*p* < 0.01) compared to the other yoghurt sauces. The ingredient results (Table 6) show that lipo-soluble rosemary extract (77.28% inhibition) could be the ingredient that provides the highest antioxidant effectiveness to the yoghurt sauce. Other ingredients presented different values from 1.62% for salt to 74.72% for the mixture of rosemary extract (water/lipo-soluble). Although Greek yoghurt is the most important ingredient, RE, used in very low proportion, is probably the ingredient that produces a strong influence on the antioxidant capacity of the final product. As for the commercial sauces analysed (Figure 1), only three (S3, S4 and S5) showed similar values to the RLE yoghurt sauce and the rest of the commercial sauces had decreased the antioxidant value lower by about 50% compared to the RLE yoghurt sauce. 

The Rancimat test is used to determine the oxidative stability of fats. In addition, it allows to evaluate the possible deterioration that handling or industrial processing can produce on fats, which could result in a decrease in their oxidative stability [30]. The highest PF value (Table 5) was observed in RLE and RWLE yoghurt sauce, where an increase in oxidative protection of around 45% was shown compared to the Control sauce, with significant differences (*p* < 0.001) compared to the other sauces. This protection is also observed in the IN5 + RWLE yoghurt sauce with an increase in oxidative protection of 20% (*p* < 0.05). Of ingredients analysed, the RLE obtained the best PF value (2.04) with respect to the rest of the ingredients, providing effective protection to the final product. As regards commercial sauces (Figure 1), all showed PF higher than 1 (between 1.09 and 1.41), showing S4, S5 and S6 yoghurt sauces with values similar to yoghurt sauce supplemented with RLE.

At the fourth week of linoleic acid autoxidation (Table 5), RLE added to the yoghurt sauce doubled protection against linoleic acid oxidation, with significant differences compared to the other sauces (*p* < 0.001). In addition to the protective effect of RLE, other ingredients could be involved in the high protection against linoleic oxidation, in decreasing order, honey, Greek yoghurt, Dijon mustard (Table 6). Nevertheless, the inulin and sunflower oil ingredients do not protect in this assay. As for commercial sauces, although all contain antioxidant additives in their composition, only S6 sauce (with olive oil) showed good protection against linoleic acid oxidation during the 4 weeks (data not shown).

## 4. Discussion

The effect of inulin on physicochemical and antioxidant properties in yoghurt sauces was studied.

The substitution of SO by IN (5%) in yoghurt sauces reduces fat content by 30%. It also lowers the caloric load. A low fat/calorie product is obtained compared to what is available in the market. Our results are in accordance with Hashemi et al. (2015) [16] who investigated the effects of replacing 5% fat for inulin on the physicochemical characteristics of ice cream and concluded that inulin may be used to manufacture a low-calorie ice cream. Similarly, Guggisberg et al. (2009) [31] investigated the inulin effect added to yoghurt with different levels of fat (0.2%, 1%, 2% and 3.5%) on physicochemical properties and observed that total solids ranged from 10.7 to 17.4, respectively. Results showed that inulin can be technologically and nutritionally interesting when mixed in yoghurts (whole and low-fat). Moreover, Guardeño et al. (2013) [32] analysed white sauces made with soy protein and gluten-free starches substituting the oil for inulin, with no significant differences (*p* < 0.05) found in texture and flavour. Likewise, in other dairy foods, Meyer et al. (2011) [33] used inulin to replace fat and obtain low-fat cheeses. The fat-substituting property of inulin is based on its ability to stabilise the structure of the aqueous phase, which creates an improved creaminess. Adding 5% inulin to the low-fat cheese resulted in a significantly lower hardness compared to the control cheese. Inulin has the property of retaining water; thus decreasing syneresis in foods such as sauces, among others [13,34].

The influence of different inulin concentrations on pH did not significantly affect values. As with our results, Guggisberg et al. (2009) [31] concluded that pH values did not significantly affect yoghurts with higher inulin additions. In general, sauces are composed of a vegetable oil or fat-free oil substitute base and a low pH, salt-containing aqueous phase. Vegetable oils and fat-free oil substitutes do not contribute to the pH of sauces [3].

Replacement of SO with IN resulted in a slight yellow that consumers might find difficult to detect sensorially. Inulin powder is whiter than sunflower oil; however, the resulting colour of this type of colloidal system also depends on their emulsifying and foaming properties. When fat is reduced in fat emulsion-based products there is a decrease in lightness, which may alter consumer perception of product quality (e.g., the desirable “creamy” appearance) [35]. Our results coincide with those of Guardeño et al. (2012) [14] who detected colour difference values in white sauces made with inulin not considered detectable by the human eye. In addition, Herranz et al. (2019) [1] supplemented fibre-enriched white sauces using apple, potato and microcrystalline cellulose such as milk and starch replacer. Similar results on fibre-enriched white sauces were darker in colour, with significantly greater yellowness values and the lowest lightness.

Furthermore, the effect of inulin did not significantly affect the antioxidant properties, except in the linoleic acid autoxidation assay. The highest values of inhibition of linoleic acid oxidation of the yoghurt sauces with inulin could be explained by the lower content of sunflower oil which produces a high linoleic acid oxidation, perhaps due to its high content of polyunsaturated fatty acids which oxidise easily. Moreover, when inulin is evaluated individually as an ingredient, although the protection against linoleic acid oxidation is lower than other ingredients, there could be a synergistic effect with the rest of the ingredients in the food matrix. Data regarding antioxidant activity in inulin are contradictory. According to Choudhary et al. (2019) [36], supplementation with inulin in soy milk improved antioxidant activity during storage in refrigeration conditions. Yadav et al. (2012) [37] supplemented a soy yoghurt and observed the highest antioxidant capacity when milk powder, inulin and strawberry pulp were added. However, the opposite effects were observed in other studies, where Tomas et al. (2018) [38] investigated the effect of the addition of inulin (5 and 10%) on the total antioxidant capacity, the content of α-tocopherol and carotenoids in tomato sauces decreasing significantly. The bioaccessibility of bioactive compounds was negatively affected by the addition of inulin in sauces.

The effect of RE on the physicochemical and antioxidant properties in yoghurt sauces has also been studied. The RE (RWE and RLE) used have a high polyphenolic content, compared to those tested. RLE alone or mixed (RWLE) increased antioxidant capacity in yoghurt sauces.

Taking into account the polyphenol content of the RE determined, RWE was rich in rosmarinic acid, salvanic acid, luteolin-7-glucoside derivative and luteolin-7-O-glucoronide, while the most abundant polyphenols of RLE were rich in carnosol, carnosic acid, carnosic acid derivative, rosmarinic acid and carnosol derivative. According to the results obtained, yoghurt sauces with RWE and RLE showed different antioxidant activity in a lipid environment (higher antioxidant capacity with the lipid-soluble extract). This might be due to the presence of carnosol which has been shown to be a lipo-soluble diterpene with a high antioxidant capacity, evaluated in emulsified lipid systems, protecting fatty acids and triglycerides against oxidation. Likewise, Loussouarn et al. (2017) [39] confirmed that carnosic acid reacts directly with lipid radicals and blocks the chain lipid peroxidation process. Carnosol is as efficient as carnosic as an antioxidant and lipid protector. Both compounds have a single aromatic ring with two OH groups that can serve as H+ donors. In addition, vicinal OH groups can chelate prooxidative metals, thereby preventing oxidation. On the other hand, rosmarinic acid inhibits lipid peroxidation induced by a hydrophilic radical generator, as a function of concentration [40]. This polyphenol has two aromatic rings, each with two OH groups capable of donating H+ and chelating metals [6]. It should be noted that other water-insoluble compounds, such as carotenoids, tocopherols, phytosterols, etc., could also provide antioxidant activity [28]. In fact, yoghurt sauces with RWE did not show significant differences with the Control sauce. It should be noted that the main ingredient of the prepared sauces is Greek yoghurt which showed very good antioxidant activity. In this regard, several studies also reported the generation of potent antioxidant peptides from milk proteins. It can be deduced that the peptides generated had acted as hydrogen or electron donors and may have reacted with free radicals giving rise to stable products [41]. There are different factors that can influence the effectiveness of an antioxidant compound, such as its composition, the combination with other compounds and its location where the oxidation reaction takes place [42].

Previous studies on the antioxidant properties of RE in oxidative stability have shown that RE added to butter at different concentrations produced higher DPPH radical scavenging than control samples [7]; similar antioxidant capacity was observed by Khalil et al. (2017) [8] adding 250 ppm RE to butter by using TBARS determination; in accordance with our results, Rahila et al. (2017) [6] observed that in ghee with added RE there was an enhancement in antioxidant capacity by longer induction period (Rancimat test) than that of control; other authors demonstrated that RE inhibited lipid oxidation in cow milk enriched with fish oil [9]. Likewise, the present study also showed that yoghurt sauce supplemented with inulin (IN5) and rosemary mixture (RWLE) increased antioxidant activity in a lipid environment.

The influence of different ER on yoghurt sauce did not significantly affect the proximate composition. Similar to our results, Rahila et al. (2017) [6] observed that the physicochemical and sensory characteristics were the same when ghee was enriched with RE. However, in our study, only the yellowness of yoghurt sauces was affected significantly by adding rosemary extract. This same effect was found in the sauce when combining the inulin (IN5) with the rosemary mixture (RWLE).

## 5. Conclusions

The replacement of SO by IN (5%) in yoghurt sauces decreased fat content by around 30% and showed roughly 15% lower calories, thereby obtaining healthier sauces. The combination of inulin and RE resulted in a slightly darker and yellow yoghurt sauce.

RLE improved lipid peroxidation inhibition of yoghurt sauces by 44%. In addition, the RLE in yoghurt sauce increased oxidative protection (Rancimat test) of around 45%, while in yoghurt sauce with inulin and RLE the increase in oxidative protection was 20%. RLE added to the yoghurt sauce doubled protection against linoleic acid oxidation. In summary, the addition of RLE, with a high polyphenolic content, was particularly effective in improving antioxidant activity in a lipidic environment.

IN and RE in yoghurt sauce are interesting from a nutritional viewpoint (replacement of lipids by dietary fibre) and technological interest (foaming agent), and RLE can be used combined with RE of high polyphenol content as a potential functional ingredient capable of stabilising sauces against oxidation.

## Figures and Tables

**Table 1:** Polyphenol content (mg/g) of rosemary extracts.

	RWE	RLE
**Phenolic acids**		
Salvianic acid	16.88	2.26
Rosmarinic acid	76.77	16.12
**Flavonoids**		
Luteolin-7-*O*-glucoronide	10.77	1.73
Luteolin-glucoside derivative	11.88	2.48
Luteolin glucoside	7.62	2.47
Cirsimaritin	2.64	2.37
Genkwanin	3.33	2.47
Hesperidin	11.25	2.24
**Diterpenes**		
Carnosol derivative	<LQ	2.82
Carnosol	0.38	35.17
Carnosic acid derivative (1)	<LQ	17.50
Carnosic acid	4.13	20.75
Carnosol derivative (2)	<LQ	11.97
**Total content**	**145.65**	**120.28**

RWE: rosemary water extract; RLE: rosemary lipo extract. LQ: 0.01 mg/g.

**Table 2:** Proximate composition (g/100 g) of yoghurt sauces made with inulin and/or rosemary extract.

	Moisture	Ash	Protein	Fat		Carbohydrate		Energy kcal/kJ	
Yoghurt Sauce	M	M	M	M		M		M	
Control	69.1	1.0	2.6	17.4	a	9.8	c	206/863	a
IN									
IN2	68.9	1.2	2.6	13.7	b	13.6	b	188/789	ab
IN5	69.2	1.1	2.7	11.9	c	14.9	a	178/746	b
RE									
RWE	68.9	1.1	2.7	17.5	a	9.9	c	207/867	a
RLE	69.1	1.1	2.6	17.6	a	9.6	c	207/866	a
RWLE	69.1	1.1	2.5	17.7	a	9.7	c	207/869	a
IN5 + RWLE	69.0	1.1	2.6	12.0	c	15.3	a	179/751	b
SEM	0.182	0.059	0.284	0.564		0.551		2.859	
Effects (*p*-values)									
IN	NS	NS	NS	***		**		***	
RE	NS	NS	NS	NS		NS		NS	
IN5 + RWLE	NS	NS	NS	**		**		NS	

Abbreviations: M: mean; SEM: standard error of mean. Formulations: IN: inulin × sunflower oil; IN2: 2% inulin; IN5: 5% inulin; RE: rosemary extracts; RWE: rosemary water extract (300 mg/kg); RLE: rosemary lipo extract (300 mg/kg); RWLE: RWE + RLE (300 mg/kg at 1:1 *w*:*w*); a, b, c: formulation effects (*p* < 0.05; Tukey test); significance levels: *** *p* < 0.001; ** *p* < 0.01; NS *p* > 0.05.

**Table 3:** Ingredients and nutritional declarations for six commercial yoghurt sauces (g/100 g).

Commercial Yoghurt Sauces	Ingredients	Protein	Fat	Carbohydrate	Salt	Energy (kcal/kJ)
1	Refined sunflower oil, alcohol vinegar, lactose-free yoghurt (6%), sugar, modified corn starch, xanthan gum, guar gum and pectin, lactic acid, pea protein, potassium sorbate, tocopherol-rich extract, flavourings, natural garlic and spices.	0.7	23	9.3	0.05	247/1023
2	Sunflower oil, alcohol vinegar, onion, sugar, dairy preparation 2.6%, salt, modified potato starch, mustard (alcohol vinegar, mustard seeds, glucose–fructose syrup, curcumin salt, natural flavouring), egg yolk powder, yoghurt powder 0.3%, xanthan gum, dehydrated chives, citric acid, parsley, natural coriander flavouring.	0.6	35	6	0.97	343/1414
3	Sunflower oil, yoghurt (12%), sugar, corn starch, mustard, wine vinegar, pasteurised egg yolk, salt, milk powder, lactic acid, potassium sorbate and sodium benzoate, spices, flavour and antioxidant (E385).	0.8	23.9	8.7	1.0	257/1064
4	Sunflower oil, pasteurised yoghurt (15%), vinegar, mustard (vinegar, mustard seed, salt, curcumin and sucralose), salt, egg yolk, modified starch, lactic acid, lactose, flavour, xanthan gum, spices, potassium sorbate and sodium benzoate, tocopherol-rich extract, EDTA and sucralose.	4.6	20	7.7	2.5	228/946
5	Soybean oil 40%, wine vinegar, sugar, fermented skimmed milk powder 3%, egg yolk, salt, modified starch, lactic acid, potassium sorbate, xanthan gum, spice, chives, dill, tocopherol, disodium and calcium EDTA.	1.5	41	7.5	2.0	406/1676
6	Greek yoghurt 70%, cucumber 14%, olive oil 14%, mint 0.2% garlic, pepper, salt, sugar, citric acid, E415, E202, E211.	4.9	15.6	6.8	0.7	188/784

**Table 4:** CIELab colour and pH values of yoghurt sauces made with inulin and/or rosemary extracts.

	CIELab Colour					pH
Yoghurt Sauces	L *		a *	b *		
	M		M	M		M
Control	87.74	a	−1.22	11.72	c	4.22
IN						
IN2	87.53	a	−1.28	11.72	c	4.17
IN5	86.97	b	−1.41	12.50	b	4.20
RE						
RWE	87.70	a	−1.18	12.24	b	4.21
RLE	87.70	a	−1.43	12.45	b	4.17
RWLE	87.51	a	−1.36	12.36	b	4.13
IN5 + RWLE	86.52	c	−1.47	13.04	a	4.21
SEM	0.097		0.090	0.091		0.031
Effects (*p*-values)						
IN	**		NS	***		NS
RE	NS		NS	***		NS
IN5 + RWLE	**		NS	***		NS

Abbreviations: M: mean; SEM: standard error of mean. Formulations: IN: inulin × sunflower oil; IN2: 2% inulin; IN5: 5% inulin; RE: rosemary extracts; RWE: water (300 mg/kg); RLE: lipo (300 mg/kg); RWLE: RWE + RLE (300 mg/kg at 1:1 *w*:*w*); a, b, c: formulation effects (*p* < 0.05; Tukey test); significance levels: *** *p* < 0.001; ** *p* < 0.01; * *p* < 0.05; NS *p* > 0.05.

**Table 5:** Antioxidant activity evaluated by different assays (hydroxyl radical scavenging, DPPH, lipid peroxidation, Rancimat test and inhibition of autooxidation linoleic acid) in yoghurt sauces made with inulin and/or rosemary extracts.

	OH Radical Scavenging	DPPH Radical Scavenging	Lipid Peroxidation		Rancimat		Autooxidation Linoleic Acid							
Yoghurt Sauces	% Inhibition	% Inhibition	% Inhibition		PF		% Inhibition							
	M	M	M		M		Week 1 M		Week 2 M		Week 3 M		Week 4 M	
Control	89.02	63.23	54.70	b	0.92	c	1.87	b	20.23	c	46.50	b	46.93	c
IN														
IN2	92.56	65.53	57.03	b	1.05	c	38.43	a	54.90	b	86.93	a	81.47	b
IN5	87.03	65.93	55.00	b	0.99	c	47.73	a	51.63	b	90.63	a	81.23	b
RE														
RWE	88.40	63.40	58.93	b	0.89	c	14.10	b	23.10	c	34.23	c	36.30	c
RLE	88.09	66.87	78.27	a	1.34	a	47.83	a	97.30	a	96.57	a	97.93	a
RWLE	88.47	65.07	72.87	a	1.28	ab	37.03	a	88.90	a	86.03	a	80.20	b
IN5 + RWLE	90.50	68.37	71.37	a	1.11	bc	39.43	a	85.67	a	86.17	a	84.90	b
SEM	1.495	3.126	2.741		0.067		5.925		4.095		3.428		3.517	
Effects (*p*-values)														
IN	NS	NS	NS		NS		***		**		***		***	
RE	NS	NS	***		***		***		***		***		***	
IN5 + RWLE	NS	NS	**		*		***		***		***		***	

Abbreviations: M: mean; SEM: standard error of mean. DPPH: 2,2-diphenyl-1-picrylhydrazyl; PF: protection factor. Formulations: IN: inulin × sunflower oil; IN2: 2% inulin; IN5: 5% inulin; RE: rosemary extracts; RWE: water (300 mg/kg); RLE: lipo (300 mg/kg); RWLE: RWE + RLE (300 mg/kg at 1:1 *w*:*w*); a, b, c: formulation effects (*p* < 0.05; Tukey test); significance levels: *** *p* < 0.001; ** *p* < 0.01; * *p* < 0.05; NS *p* > 0.05.

**Table 6:** Average (*n* = 3) antioxidant activity separately evaluated in each yoghurt ingredient by different assays (hydroxyl radical scavenging, DPPH, lipid peroxidation, Rancimat test and inhibition of autooxidation linoleic acid).

	OH Radical Scavenging	DPPH Radical Scavenging	Lipid Peroxidation	Rancimat	Autooxidation Linoleic Acid
Ingredients	% Inhibition	% Inhibition	% Inhibition	PF	% Inhibition Week 4
	M ± SEM	M ± SEM	M ± SEM	M ± SEM	M ± SEM
Greek yoghurt	74.87 ± 0.01	69.00 ± 7.30	57.16 ± 0.17	1.08 ± 0.01	87.38 ± 0.94
Honey	78.30 ± 0.04	55.30 ± 3.35	16.85 ± 0.02	1.00 ± 0.02	96.62 ± 0.46
Sunflower oil	-	35.00 ± 3.03	45.40 ± 0.26	-	-
Dijon mustard	42.01 ± 0.07	79.70 ± 1.80	67.11 ± 0.07	1.10 ± 0.01	82.96 ± 1.24
Salt	-	35.60 ± 1.23	1.62 ± 0.22	1.30 ± 0.01	63.55 ± 2.53
IN2	35.71 ± 0.07	42.80 ± 2.11	6.43 ± 0.05	0.97 ± 0.02	23.59 ± 4.40
IN5	63.13 ± 0.04	55.20 ± 3.44	3.45 ± 0.01	1.02 ± 0.01	5.93 ± 1.40
RWE	-	93.20 ± 2.79	65.66 ± 0.03	1.18 ± 0.00	95.77 ± 0.93
RLE	-	92.60 ± 2.00	77.28 ± 0.03	2.04 ± 0.02	92.49 ± 1.21
RWLE	-	86.90 ± 4.65	74.72 ± 0.01	1.18 ± 0.01	94.58 ± 0.82

Abbreviations: M: mean; SEM: standard error of mean; DPPH: 2,2-diphenyl-1-picrylhydrazyl; PF: protection factor. Formulations: IN: inulin × sunflower oil; IN2: 2% inulin; IN5: 5% inulin; RE: rosemary extracts; RWE: water (300 mg/kg); RLE: lipo (300 mg/kg); RWLE: RWE + RLE (300 mg/kg at 1:1 *w*:*w*).

## Data Availability

Data is contained within the article.
